# SLC40A1-mediated positive feedback loop with M1 macrophages suppresses epithelial ovarian cancer progression

**DOI:** 10.3389/fimmu.2025.1709597

**Published:** 2026-01-14

**Authors:** Guangyan Wang, Sisi Huang, Bo Yin, Fugen Shangguan, Jinghang Ma, Anyang Li, Zhexin Xia, Zhenzhen Xu, Yuheng Li, Honglei Jin, Yan Hu, Baoyou Huang

**Affiliations:** 1 Department of Gynecology, The First Affiliated Hospital of Wenzhou Medical University, Wenzhou, Zhejiang, China; 2 Department of Pathology, The First Affiliated Hospital of Wenzhou Medical University, Wenzhou, Zhejiang, China; 3 Department of Gynecology, Shanghai Key Laboratory of Maternal Fetal Medicine, Shanghai Institute of Maternal-Fetal Medicine and Gynecologic Oncology, Clinical and Translational Research Center, Shanghai First Maternity and infant Hospital, School of Medicine, Tongji University, Shanghai, China; 4 Zhejiang Key Laboratory of Intelligent Cancer Biomarker Discovery and Translation, First Affiliated Hospital of Wenzhou Medical University, Wenzhou, China; 5 Department of Information Technology, the First Affiliated Hospital of Wenzhou Medical University, Wenzhou, Zhejiang, China; 6 The First Clinical Medical College of Wenzhou Medical University, Wenzhou, Zhejiang, China; 7 Zhejiang Provincial Key Laboratory of Medical Genetics, Key Laboratory of Laboratory Medicine, Ministry of Education, School of Laboratory Medicine and Life Sciences, Wenzhou Medical University, Wenzhou, Zhejiang, China

**Keywords:** biomarker, epithelial ovarian cancer, M1 macrophage, macrophage polarization, SLC40A1

## Abstract

**Introduction:**

Ovarian cancer (OC), particularly epithelial ovarian cancer (EOC), represents one of the most lethal and aggressive gynecological malignancies. Despite advances in surgery, chemotherapy, and immunotherapy, patient survival remains poor. Identifying novel molecular targets is crucial for improving early diagnosis and developing more effective therapies.

**Methods:**

We examined the expression and immunoregulatory function of SLC40A1 in EOC using both experiments on cells and mouse orthotopic tumor models. Through integrated *in vitro* and *in vivo* studies, we systematically assessed the role of SLC40A1 in promoting M1 macrophage polarization and its relationship with tumor suppression, demonstrating that SLC40A1 enhances the response to immunotherapy.

**Results:**

SLC40A1 was found to be more highly expressed in normal ovarian tissues compared with EOC tissues, and its high expression was associated with a favorable prognosis. *In vitro*, SLC40A1 did not significantly affect tumor cell proliferation, apoptosis, or migration and invasion. However, *in vivo* experiments using mice with differing immune status demonstrated that SLC40A1 modulates the tumor immune microenvironment. Subsequent bioinformatics analyses suggested that SLC40A1 may regulate M1 macrophage polarization. Mechanistically, *in vitro* experiments confirmed that SLC40A1 regulates CXCL11 secretion, which activates the JAK2–STAT1 signaling pathway, promoting macrophage TNF-α production, which in turn upregulates SLC40A1 expression. Finally, we demonstrated that SLC40A1 enhances the response to immunotherapy.

**Discussion:**

These findings identify SLC40A1 as a key regulator of the antitumor immune response in EOC. High SLC40A1 expression is associated with enhanced macrophage-mediated tumor suppression and improved response to immunotherapy, highlighting its potential as both a prognostic biomarker and a therapeutic target.

## 1 Introduction

Epithelial ovarian cancer (EOC) is the predominant histological subtype of ovarian cancer (OC) and remains a leading cause of gynecological cancer-related mortality worldwide (1, 2). Despite advances in early detection and treatment, including surgery and chemotherapy, the prognosis for patients with advanced-stage EOC remains poor due to frequent recurrence and metastasis (3, 4). Emerging evidence indicates that the infiltration and crosstalk of immune cells within the tumor microenvironment (TME) critically influence patient outcomes (5). Accordingly, immunotherapy has gained attention as a promising strategy to improve survival in EOC (6). A deeper understanding of the interactions between ovarian tumor cells and immune components within the TME is essential for developing effective and personalized immunotherapeutic approaches (7).

Macrophages are among the most abundant immune cells infiltrating ovarian tumors and play a central role in shaping the tumor immune microenvironment (TIME) (8). They can adopt diverse functional states, with pro-inflammatory M1 macrophages exhibiting potent antitumor activity (9, 10). Tumor cells secrete a variety of signaling molecules, including cytokines and chemokines, that influence macrophage recruitment, differentiation, and activation within the TME (11). By modulating these interactions, macrophages can either enhance or suppress antitumor immunity. In addition, the spatial distribution and functional heterogeneity of macrophages within tumors have been shown to critically impact tumor progression, therapeutic responses, and patient prognosis (12). Understanding the mechanisms governing macrophage activation and their crosstalk with tumor cells is therefore essential for the development of effective immunotherapeutic strategies in EOC.

SLC40A1, also known as ferroportin, is a critical regulator of systemic iron homeostasis and plays essential roles in cellular iron export (13, 14). Beyond its classical function in iron metabolism, emerging studies have suggested that SLC40A1 can modulate immune responses, particularly by influencing macrophage activation and polarization (15, 16). However, the precise impact of SLC40A1 on macrophage function remains controversial, with some reports indicating pro-inflammatory effects, while others suggest context-dependent immunosuppressive roles (17–19). Despite these intriguing findings, the role of SLC40A1 in EOC and its interaction with tumor-infiltrating macrophages has been scarcely investigated, highlighting the need for further studies to elucidate its immunoregulatory functions in this setting.

Our study demonstrates that SLC40A1 plays a pivotal role in modulating the TIME by promoting the polarization of macrophages toward the M1 phenotype. This shift enhances macrophage-mediated antitumor activity, leading to suppressed tumor growth and improved responsiveness to immunotherapy. Mechanistically, SLC40A1 orchestrates a positive feedback loop that reinforces pro-inflammatory macrophage functions, thereby amplifying antitumor immune responses within the TME in EOC. Collectively, these findings underscore the therapeutic potential of targeting the SLC40A1–M1 macrophage axis as a novel strategy to inhibit EOC progression and improve patient outcomes.

## 2 Materials and methods

### 2.1 Clinical specimens and ethical approval

EOC tissue and normal ovary samples were collected from individuals who underwent surgical resection at the First Affiliated Hospital of Wenzhou Medical University. All clinicopathological diagnoses were independently confirmed by at least two pathologists. None of the patients had received chemotherapy or radiotherapy prior to surgery. This study was approved by the Ethics Committee of the First Affiliated Hospital of Wenzhou Medical University (Approval No. YS2022-442), and written informed consent was obtained from all participants. The study was conducted in accordance with the principles of the Declaration of Helsinki and the Council for International Organizations of Medical Sciences (CIOMS) International Ethical Guidelines for Health-related Research Involving Humans.

### 2.2 Bioinformatics analysis

As described previously (20), RNA-sequencing (RNA-seq) raw data of cancer cohorts were obtained and processed from The Cancer Genome Atlas (TCGA; https://portal.gdc.cancer.gov/), and normal ovarian tissue data were obtained from the Genotype-Tissue Expression (GTEx) public database. Microarray datasets were downloaded from the Gene Expression Omnibus (GEO; https://www.ncbi.nlm.nih.gov/geo/). The Kaplan–Meier survival analysis (http://kmplot.com/analysis/) was performed to evaluate the impact of related indicators on patient prognosis. The LinkedOmics database (http://www.linkedomics.org) was utilized to conduct co-expression and functional enrichment analyses. In addition, the CIBERSORT algorithm was applied to assess immune cell infiltration.

### 2.3 Antibodies and reagents

The antibodies were commercially available: SLC40A1 (26601-1-AP; Proteintech, Wuhan, China), glyceraldehyde 3-phosphate dehydrogenase (GAPDH) (AB2100; NCM Biotech, Suzhou, China), Ki67 (A20018; ABclonal, Wuhan, China), F4/80 (A27257; ABclonal, Wuhan, China), CD86 (83523-4-RR; Proteintech), iNOS (WL0992a; Wanleibio, Shenyang, China), JAK2 (TA6022; Abmart, Shanghai, China), Phospho-JAK2 (TB5319; Abmart, Shanghai, China), STAT1 (14994; Cell Signaling Technology, Danvers, Massachusetts, USA), Phospho-STAT1 (9167; Cell Signaling Technology, Danvers, Massachusetts, USA), CD8 (A23081; ABclonal), granzyme B (GZMB) (A2557; ABclonal), and Goat Anti-Rabbit IgG(H+L)-HRP Conjugated (LF102; Epizyme Biotech, Shanghai, China). The corresponding antibody dilutions were prepared according to the manufacturer’s instructions. Detailed information on flow cytometry antibodies and reagents is provided in **Supplementary Table S1**.

### 2.4 Cell culture and stable cell line construction

Human cell lines IOSE80 (normal epithelial ovarian cells), A2780 and SKOV3 (EOC cell line), THP-1, and 293T, as well as the murine EOC cell line ID8, were available in our laboratory and cultured according to previously described methods (21). The other human EOC cell lines, Caov3 and HEY, were newly purchased from the Cell Bank of the Type Culture Collection of the Chinese Academy of Sciences (Shanghai, China). These cells were cultured in Dulbecco's Modified Eagle Medium (DMEM) (Caov3) or Roswell Park Memorial Institute (RPMI) 1640 (HEY) containing 10% fetal bovine serum (FBS; C9050, NCM Biotech, China) and 1% penicillin/streptomycin (Gibco, Grand Island, New York, USA). The stable cell lines with SLC40A1 overexpression were generated as previously described (22). The overexpression plasmids and their corresponding negative controls (NC) were designed and synthesized by YouBio (Changsha, China).

### 2.5 Western blotting

Tissues and cells were lysed with ice-cold Radioimmunoprecipitation Assay (RIPA) buffer (WB3100, NCM Biotech, China) supplemented with protease and phosphatase inhibitors (P002, NCM Biotech, China). The lysates were incubated on ice for 30 min and centrifuged at 12,000 × *g* for 15 min at 4°C, and the supernatants were collected. Total protein concentrations were determined using the Bicinchoninic Acid (BCA) protein assay kit (Thermo Fisher Scientific, Waltham, Massachusetts, USA) according to the manufacturer’s instructions. Equal amounts of protein were mixed with loading buffer, denatured at 95°C for 5 min, and separated via Sodium Dodecyl Sulfate–Polyacrylamide Gel Electrophoresis (SDS–PAGE). Proteins were transferred onto Polyvinylidene Fluoride (PVDF) membranes, which were blocked with 5% non-fat milk in Tris-Buffered Saline with Tween 20 (TBST) for 1 h at room temperature. The membranes were incubated overnight at 4°C with primary antibodies, followed by incubation with Horseradish Peroxidase (HRP)-conjugated secondary antibodies for 1 h at room temperature. Protein bands were detected using an enhanced chemiluminescence kit and visualized using a bioanalytical imaging system.

### 2.6 Quantitative polymerase chain reaction

Total RNA from tissues and cell lines was isolated using TRIzol reagent (Takara, Japan) according to the manufacturer’s instructions. cDNA synthesis was carried out using the PrimeScript RT Master Mix kit (RK20429, ABclonal, China), and quantitative real-time PCR was subsequently performed using the RK21203 kit (ABclonal, China). GAPDH or β-actin was used as an internal reference, and relative expression levels were calculated using the 2^−ΔΔCT^ method. The primer sequences are listed in **Supplementary Table S2**.

### 2.7 Immunohistochemistry

Tissue sections were dewaxed in xylene and rehydrated through a graded ethanol series. Endogenous peroxidase activity was blocked with 3% hydrogen peroxide, followed by heat-induced antigen retrieval. Sections were incubated with primary antibodies overnight at 4°C and then exposed to the appropriate secondary antibodies for 1 h at room temperature (25°C). Immunoreactivity was visualized using 3,3′-diaminobenzidine (DAB; C520017, Sangon Biotech, Shanghai, China) in accordance with the manufacturer’s protocol, after which the slides were counterstained with hematoxylin and mounted with neutral gum.

### 2.8 5-Ethynyl-2′-deoxyuridine assay

5-Ethynyl-2′-deoxyuridine (EdU) staining was conducted using a DNA cell proliferation assay kit (CX003, Epizyme Biotech, China) following the manufacturer’s protocol. Fluorescence signals were visualized using an inverted fluorescence microscope (Nikon, Japan), and the percentage of EdU-positive cells was quantified using the ImageJ software.

### 2.9 Apoptosis assay

After digestion with Ethylenediaminetetraacetic Acid (EDTA)-free trypsin, cells were stained with Annexin V-FITC and PI (40302ES08, Yeasen Biotechnology, Shanghai, China) in 100 μL binding buffer and incubated for 30 min in the dark. Fluorescence signals were then detected via flow cytometry, with 1 × 10^4^ cells acquired for each sample.

### 2.10 Transwell assays

The migration and invasion abilities of EOC cells were evaluated using 24-well Transwell chambers (8.0-μm pore size; Corning, New York, USA). For invasion assays, membranes were pre-coated with Matrigel (D23016-0010, D1 Medical Technology, Hangzhou, China), whereas for migration assays, uncoated membranes were used. Cells were seeded in the upper chamber with 200 μL serum-free DMEM, and the lower chamber contained 600 μL medium supplemented with 10% FBS. After 24 h, non-migrated cells on the upper surface were removed, and the migrated or invaded cells were fixed with paraformaldehyde and stained with 0.1% crystal violet. Images were captured under an inverted microscope, and cell counts were quantified using the ImageJ software (USA).

### 2.11 Animal experiment

All *in vivo* experiments were conducted in accordance with the guidelines of the National Institutes of Health for the care and use of laboratory animals and were approved by the First Affiliated Hospital of Wenzhou Medical University (Approval No. WYYY-AEC-YS-2022-0198). For the orthotopic tumor model, 6-week-old female Balb/C-nude and C57BL/6 mice were used for tumor transplantation. For *in vivo* tumor growth assays, 1 × 10^6^ ID8 cells (20 µL; NC vs. OE-SLC40A1) were mixed 1:1 with Matrigel (20 µL, D23016-0010, D1 Medical Technology, Hangzhou, China) and injected into the ovarian bursa to establish orthotopic tumors. Tumor growth was monitored, and 30 days post-injection, mice were anesthetized with isoflurane and euthanized by cervical dislocation. Tumors were subsequently collected for further analyses.

### 2.12 Flow cytometry

Consistent with previous studies, tumor samples were dissociated into single-cell suspensions and stained with antibodies for 30 min at room temperature (23). Following Phosphate-Buffered Saline (PBS) washes and centrifugation at 1,000 rpm for 5 min, cells were resuspended in 100 μL PBS and analyzed via flow cytometry (FACSCalibur, BD Biosciences, NJ, USA). M0 macrophages were identified as CD45^+^CD11b^+^F4/80^+^.

To characterize THP-1-derived macrophages, M1 and M2 phenotypes were detected via flow cytometry. M1 macrophages were identified as CD11b^+^CD86^+^, while M2 macrophages were defined as CD11b^+^CD206^+^. Cells were incubated with the respective fluorescently labeled antibodies at room temperature for 30 min in the dark, washed with PBS, and analyzed using a flow cytometer (FACSCalibur, BD Biosciences, NJ, USA).

### 2.13 Immunofluorescence staining

Paraffin-embedded tumor sections were deparaffinized in xylene and rehydrated through a graded ethanol series. Antigen retrieval was performed using heat-induced epitope retrieval, and non-specific binding was blocked with 5% bovine serum albumin (BSA) for 30 min at room temperature. Sections were then incubated overnight at 4°C with primary antibodies against F4/80 and CD86. After washing with PBS, sections were incubated with species-appropriate fluorescent secondary antibodies for 1 h at room temperature in the dark. Nuclei were counterstained with 4′,6-Diamidino-2-Phenylindole (DAPI), and images were acquired using a confocal fluorescence microscope.

### 2.14 Preparation of conditioned medium

A2780 cells stably overexpressing SLC40A1 were seeded in 10-cm dishes and cultured in complete medium until reaching approximately 70%–80% confluence. The cells were then washed twice with PBS and incubated in serum-free RPMI 1640 medium for 24 h. The supernatants were collected, centrifuged at 1,000 × *g* for 10 min to remove cell debris, and subsequently filtered through a 0.22-μm membrane. The resulting supernatants were designated as conditioned medium (CM) from SLC40A1-overexpressing A2780 cells and stored at −80°C until further use.

### 2.15 *In vivo* PD-1 treatment

Six-week-old female C57BL/6 mice were orthotopically implanted by injecting 1 × 10^6^ tumor cells mixed 1:1 with Matrigel (D23016-0010, D1 Medical Technology, Hangzhou, China) into the ovarian bursa. Anti-PD-1 antibody (200 µg per mouse, intraperitoneally; S0B0594, STARTER) or an isotype control was administered on days 10, 15, and 25 after orthotopic implantation, with mice randomly assigned to each treatment group. Thirty days after implantation, mice were anesthetized with isoflurane and euthanized by cervical dislocation, and tumors were harvested for further analyses.

### 2.16 Enzyme-linked immunosorbent assay

Secretion of CXCL11 into the culture supernatant from cells was detected using the enzyme-linked immunosorbent assay (ELISA) kits (SYP-H0030, UpingBio, Hangzhou, China) according to the manufacturer’s instructions.

### 2.17 Quantification and statistical analysis

Statistical analyses were conducted using R (v4.2.2) or GraphPad Prism (v9). Cell numbers were quantified with ImageJ, and flow cytometry data were analyzed using FlowJo (v10). Further details are provided in the figure legends. A p-value <0.05 was considered statistically significant.

## 3 Results

### 3.1 Upregulated SLC40A1 is associated with favorable prognosis in patients with EOC

**Figure 1:**
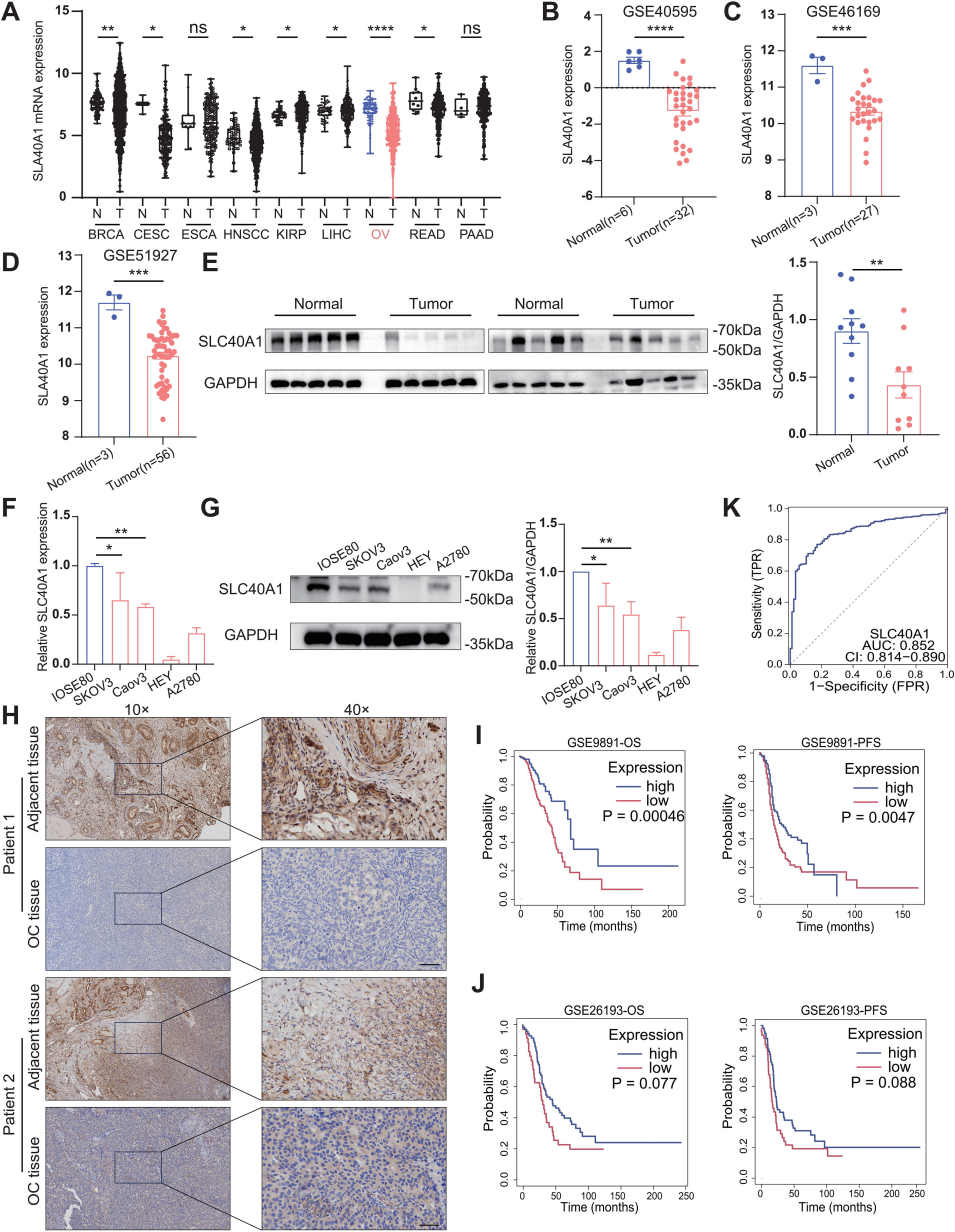
Upregulated SLC40A1 is associated with favorable prognosis in patients with epithelial ovarian cancer (EOC). **(A)** Comparison of SLC40A1 expression levels between cancer and normal tissues across various cancer types shown in The Cancer Genome Atlas (TCGA) database. **(B–D)** The mRNA expression of SLC40A1 in ovarian cancer (OC) and normal tissues was compared across three distinct Gene Expression Omnibus (GEO) datasets (GSE40595, GSE46169, and GSE51927). **(E)** The protein expression levels of SLC40A1 in EOC tissues and normal ovaries were assessed by Western blotting (WB) (left) and quantitatively analyzed (right) using ImageJ software (n = 10). **(F, G)** Comparison of SLC40A1 expression at the mRNA **(F)** and protein **(G)** levels in normal ovarian epithelial cell line (IOSE80) and EOC cell lines (SKOV3, Caov3, HEY, and A2780). **(H)** Immunohistochemistry (IHC) was employed to evaluate the expression of SLC40A1 in tumor tissues and adjacent non-tumor tissues. Scale bar, 200 µm (right). **(I, J)** The GSE9891 **(I)** and GSE26193 **(J)** datasets were analyzed to examine the effects of SLC40A1 levels on overall survival (OS) and progression-free survival (PFS) of patients. Statistical analysis was performed using the log-rank test. **(K)** The area under the curve (AUC) indicates the accuracy of SLC40A1 as a biomarker. Data are presented as the means ± standard error of the mean (SEM) **(A–E)** or standard deviation (SD) **(F, G)**. Statistical analysis was performed using unpaired two-sided Student’s t-test. *p < 0.05, **p < 0.01, ***p < 0.001, and ****p < 0.0001.

We initially analyzed the expression difference of SLC40A1 in pan-cancer from the TCGA and GTEx databases. We observed that, compared to other cancer types, the difference in SLC40A1 expression between EOC and normal samples was particularly pronounced, and its expression was higher in normal tissues (**Figure 1A**, **Supplementary Table S3**). Subsequently, we used publicly available datasets beyond TCGA to examine SLC40A1 expression in human OC. Analyses of the GEO series further revealed that SLC40A1 expression is upregulated in human normal tissues (**Figures 1B–D**). Then, we assessed SLC40A1 expression in human EOC and normal tissues obtained from the First Affiliated Hospital of Wenzhou Medical University. Our analysis also revealed that normal tissues exhibited considerably higher SLC40A1 protein expression (**Figure 1E**). The results from the cell lines were consistent with these findings. We found that the expression of SLC40A1 in the normal ovarian epithelial cell lines compared with EOC cell lines was higher at both RNA and protein levels (**Figures 1F, G**). In addition, immunohistochemistry (IHC) analyses consistently highlighted a marked increase in SLC40A1 expression in adjacent tissues (**Figure 1H**).

Lastly, the Kaplan–Meier survival analysis across multiple cohorts showed that patients with OC with higher SLC40A1 levels had longer survival times, and subsequent Receiver Operating Characteristic (ROC) curve analysis identified SLC40A1 as an independent prognostic factor for OC patients (**Figures 1I–K**). We further evaluated the prognostic value of SLC40A1 using the Gene Expression Profiling Interactive Analysis, Human Protein Atlas, OncoLnc, and TIMER2.0 databases, and we found that SLC40A1 was strikingly elevated in normal tissues compared with OC tissues, with increased expression associated with better prognoses of OC patients (**Supplementary Figures S1A–E**). These findings suggest that SLC40A1 is downregulated in OC, and its upregulation may serve as a marker of a favorable prognosis.

### 3.2 SLC40A1 overexpression shows limited influence on EOC cell phenotypic behavior

**Figure 2:**
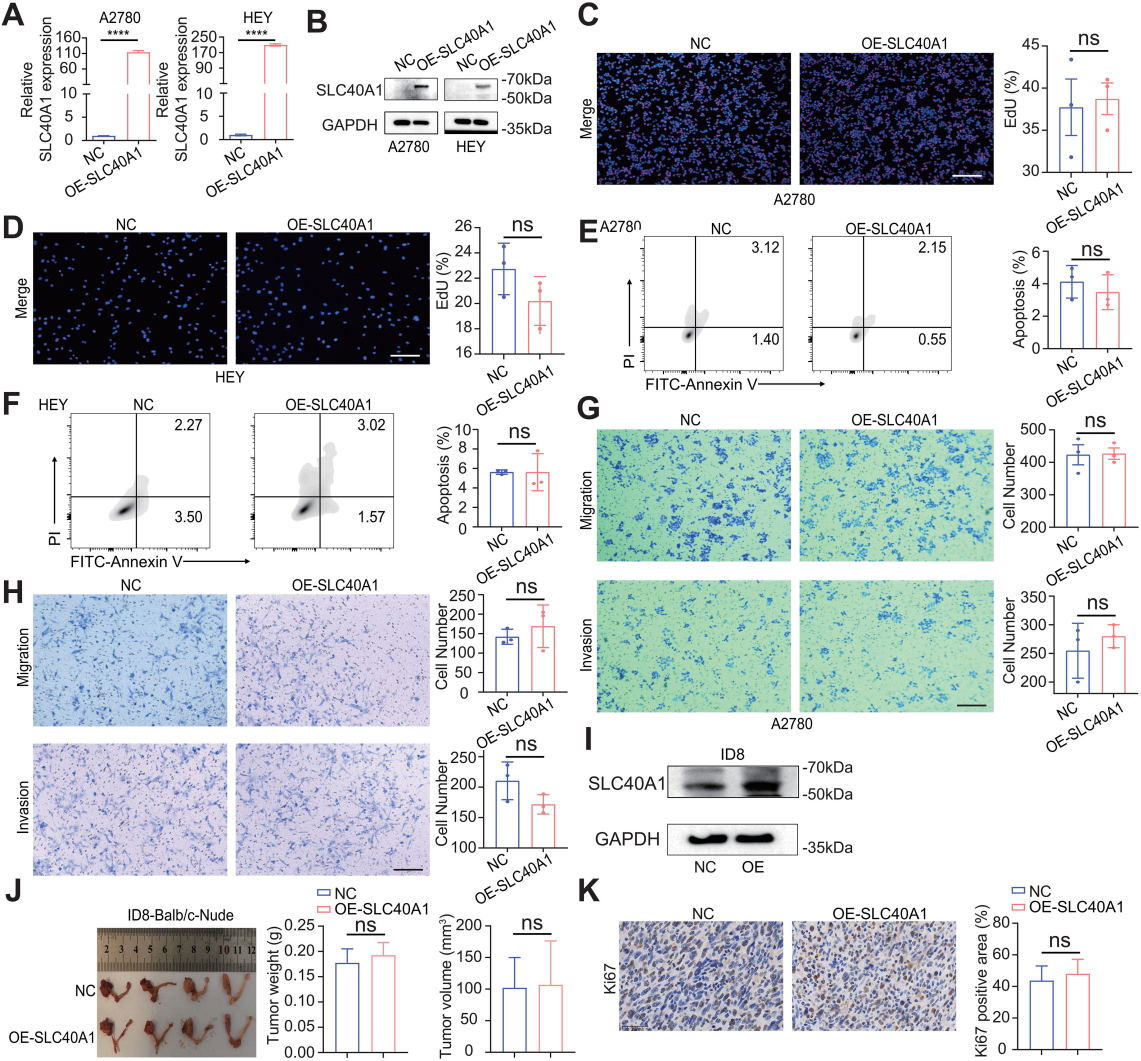
SLC40A1 overexpression shows limited influence on epithelial ovarian cancer (EOC) cell phenotypic behavior. **(A)** qPCR showed the mRNA overexpression efficiency of SLC40A1 on A2780 (left) and HEY (right) compared with negative control (NC) group. **(B)** Western blotting (WB) confirmed the protein overexpression efficiency of SLC40A1 in A2780 (left) and HEY (right). Representative WB images are shown. **(C, D)** The proliferation of A2780 **(C)** and HEY **(D)** cells, as indicated by treatments, was evaluated using 5-ethynyl-2′-deoxyuridine (EdU) assays. Scale bar, 100 μm. Representative cell images are shown. **(E, F)** The apoptosis of A2780 **(E)** and HEY **(F)** cells, as described treatments, was studied via flow cytometry. Representative results are shown. **(G, H)** The migration and invasion of A2780 **(G)** and HEY **(K)** cells according to the treatments applied were demonstrated using Transwell assays. Scale bar, 100 μm. Representative cell images are shown. **(I)** WB confirmed the protein overexpression efficiency of SLC40A1 in ID8 cell line. Representative WB images are shown. **(J)** The images of orthotopic tumors of the ID8 cells (left, NC versus OE-SLC40A1) in Balb/c-Nude mice (n = 4 mice per group) are shown, and tumor weight (middle) and tumor volume (right) of two groups were compared. **(K)** The Ki67 was evaluated by immunohistochemistry) (IHC in tumor tissues of tumor-bearing mice (left) and quantification of the percentage of Ki67-positive staining areas in tumor-bearing tissues (right) (n = 3). All panels are of the same magnification. Scale bar, 100 μm. Data are presented as mean ± SD, and statistical analyses were performed using unpaired two-sided Student’s t-test (**A, C–H, J–K**; n = 4 for panel **J** vs. n = 3 for the others). ns, not significant; ****p < 0.0001.

To investigate the effects of SLC40A1 on the malignant phenotypes of EOC, we examined its impact on cell phenotypic behavior both *in vitro* and *in vivo*. Given the low expression of SLC40A1 in tumor cells, we chose to overexpress SLC40A1 in the HEY and A2780 cell lines (NC versus OE-SLC40A1). To validate the efficiency of SLC40A1 overexpression, we performed quantitative polymerase chain reaction (qPCR) and Western blotting (WB) analyses, both confirming a significant upregulation in SLC40A1 expression (**Figures 2A, B**). To assess the impact of SLC40A1 on EOC cell proliferation, we conducted EdU assays, which revealed no differences in cell proliferation between the SLC40A1 overexpressed group compared with control cells (**Figures 2C, D**). Additionally, apoptosis assays showed similar results, which suggests that SLC40A1 overexpression did not affect cell apoptosis capacity *in vitro* (**Figures 2E, F**). Results of Transwell assays to measure cell migration and invasion displayed no differences in SLC40A1 overexpression groups compared with control cells (**Figures 2G, H**). To investigate the role of SLC40A1 *in vivo*, we first established an ID8 cell line stably overexpressing SLC40A1 and orthotopically implanted it into the ovaries of mice (**Figure 2I**). The results showed that SLC40A1 overexpression exerted little influence on tumor growth, as neither tumor size nor tumor weight differed significantly between the overexpression and control groups (**Figure 2J**). Consistently, the expression of Ki67 in tumor tissues also showed no significant change (**Figure 2K**). These results indicate that SLC40A1 overexpression had little effect on EOC cell phenotypic behavior *in vivo* and *in vitro*.

### 3.3 SLC40A1 reshaped the immune microenvironment in EOC

To further investigate the *in vivo* role of SLC40A1, we performed co-expression analysis using the LinkedOmics database. Spearman’s correlation identified genes significantly associated with SLC40A1 (**Supplementary Figure S2A**), and we visualized the top 50 positively or negatively correlated genes in a heatmap (**Supplementary Figures S2B, C**). Gene Ontology analysis revealed that SLC40A1 is associated with myeloid cell activation, leukocyte activation, and immune effector processes (**Supplementary Figure S2D**), suggesting a potential role in shaping the TIME.

**Figure 3:**
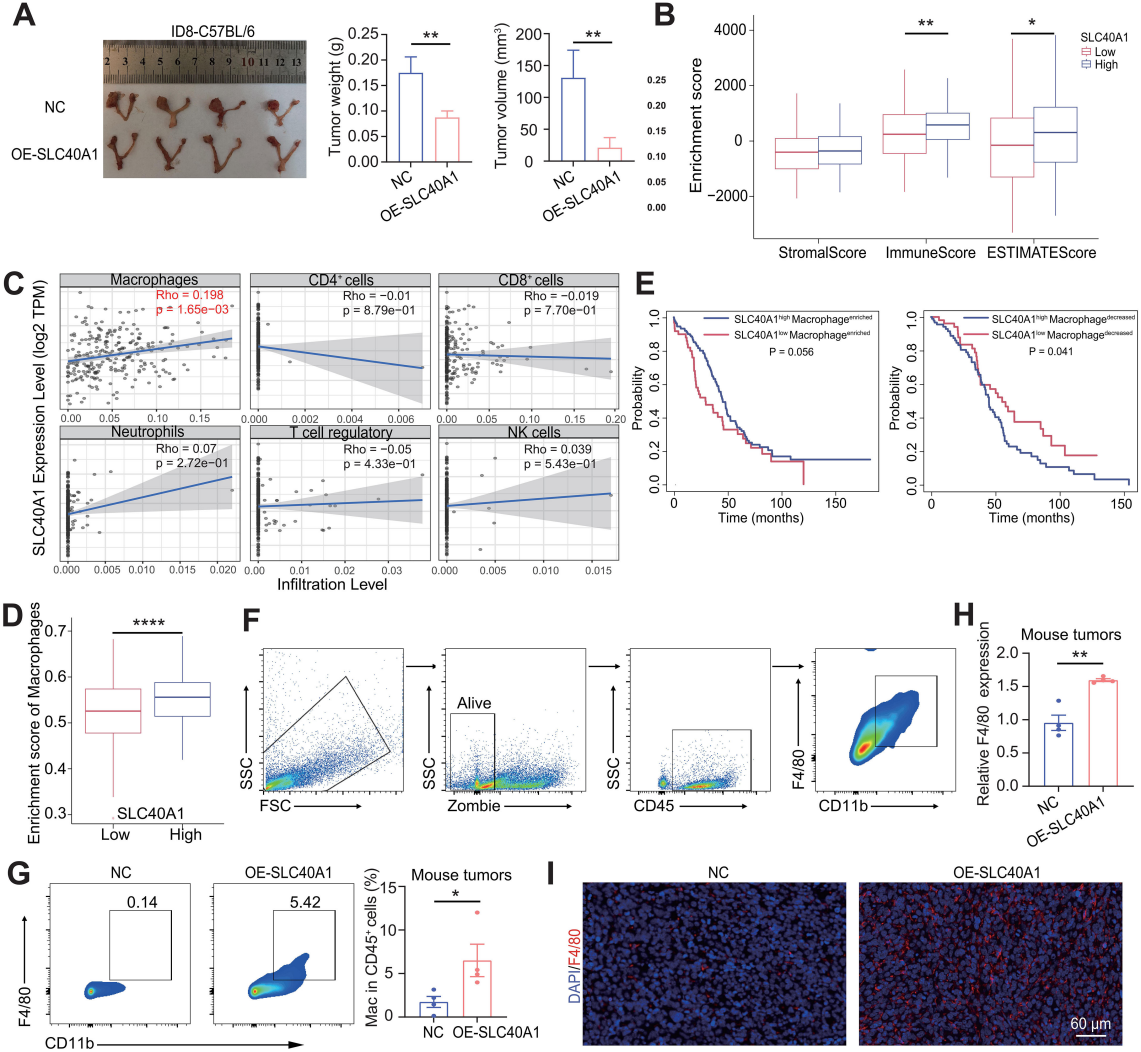
SLC40A1 modulated the immune microenvironment in epithelial ovarian cancer (EOC). **(A)** The images of orthotopic tumors of the ID8 cells [left, negative controls (NC) versus OE-SLC40A1] in C57BL/6 mice (n = 4 mice per group) are shown, and tumor weight (middle) and tumor volume (right) of two groups were compared. **(B)** Correlation between StromalScore, ImmuneScore, ESTIMATEScore, and SLC40A1 expression in TCGA-OV patients. Statistical analysis was performed using the Wilcoxon rank-sum test. **(C)** Correlation analysis between SLC40A1 and macrophage, CD4^+^ cells, CD8^+^ cells, neutrophils, T-cell regulatory, and NK-cell infiltration based on the TIMER database. **(D)** Correlation between enrichment score of macrophages and SLC40A1 expression via single-sample Gene Set Enrichment Analysis (ssGSEA) algorithm in TCGA-OV patients. Statistical analysis was performed using the Wilcoxon rank-sum test. **(E)** TCGA-OV data were classified into high macrophage and low macrophage infiltration groups. The SLC40A1 expression was used as a marker to predict the overall survival (OS) of the cohort. Statistical analysis was performed using the log-rank test. **(F)** Flow cytometry gating strategy of macrophages. Following gating to live cells by Zombie fixable viability stain, infiltrating immune cells were gated for CD45^+^ cells. Macrophages were identified as CD45^+^F4/80^+^CD11b^+^. **(G)** In orthotopic ovarian tumor model, the proportions of macrophages in tumors were analyzed (left) and quantified (right). **(H)** Detection of F4/80 mRNA expression levels in orthotopic ovarian tumor models (NC and OE-SLC40A1 groups). **(I)** Representative immunofluorescence (IF) images showing F4/80 expression in ovarian tumor models (NC and OE-SLC40A1 groups; n = 3). Scale bar, 60 μm. Data are presented as mean ± SD, and statistical analyses were performed using unpaired two-sided Student’s t-test [**(A, H, G)**; n = 4]. *p < 0.05, **p < 0.01, and ****p < 0.0001.

Based on these findings, we established an immunocompetent syngeneic orthotopic model by implanting ID8 cells with stable SLC40A1 overexpression into the ovarian bursa of C57BL/6 mice. Unexpectedly, SLC40A1 overexpression exhibited distinct effects in C57BL/6 mice compared with nude mice, as tumors derived from SLC40A1-overexpressing cells displayed smaller mass and volume than those in the control group (**Figure 3A**). This observation implies that SLC40A1 exerts its influence on tumor progression primarily through immune modulation rather than intrinsic tumor cell growth. To further validate our hypothesis, we applied the ESTIMATE algorithm to the TCGA-OV cohort and found that tumors with high SLC40A1 expression exhibited higher ImmuneScore and ESTIMATEScore (**Figure 3B**). Subsequently, using the TIMER database, we analyzed the association between SLC40A1 expression and major immune cell populations in the OC microenvironment, including macrophages, CD4^+^ T cells, CD8^+^ T cells, neutrophils, regulatory T cells, and NK cells. We observed a significant positive correlation between SLC40A1 expression and macrophage infiltration (**Figure 3C**). Consistently, single-sample Gene Set Enrichment Analysis (ssGSEA) confirmed that higher SLC40A1 expression was accompanied by elevated macrophage enrichment scores in TCGA-OV samples (**Figure 3D**). Importantly, the stratification of patients based on macrophage infiltration revealed that high SLC40A1 expression was significantly associated with favorable OS in the high-infiltration group, whereas it predicted poor prognosis in the low-infiltration group (**Figure 3E**). These findings suggest that SLC40A1 may exert tumor-suppressive effects primarily through macrophage-related immune responses.

Subsequently, we validated these findings in the murine tumor samples mentioned above (**Figure 3A**). Flow cytometry analysis was performed to assess the proportion of macrophages between the two groups (**Figure 3F**). We found that tumors derived from SLC40A1-overexpressing cells exhibited an increased proportion of CD45⁺CD11b⁺F4/80⁺ macrophages and F4/80 expression (**Figures 3G, H**). Consistently, immunofluorescence staining of tumor tissues further confirmed elevated F4/80 expression in the SLC40A1-overexpressing group (**Figure 3I**). Taken together, these results demonstrate that SLC40A1 reshapes the immune microenvironment in EOC, primarily by promoting macrophage infiltration.

### 3.4 SLC40A1 drives M1 macrophage polarization via the CXCL11–CXCR3 axis in EOC

**Figure 4:**
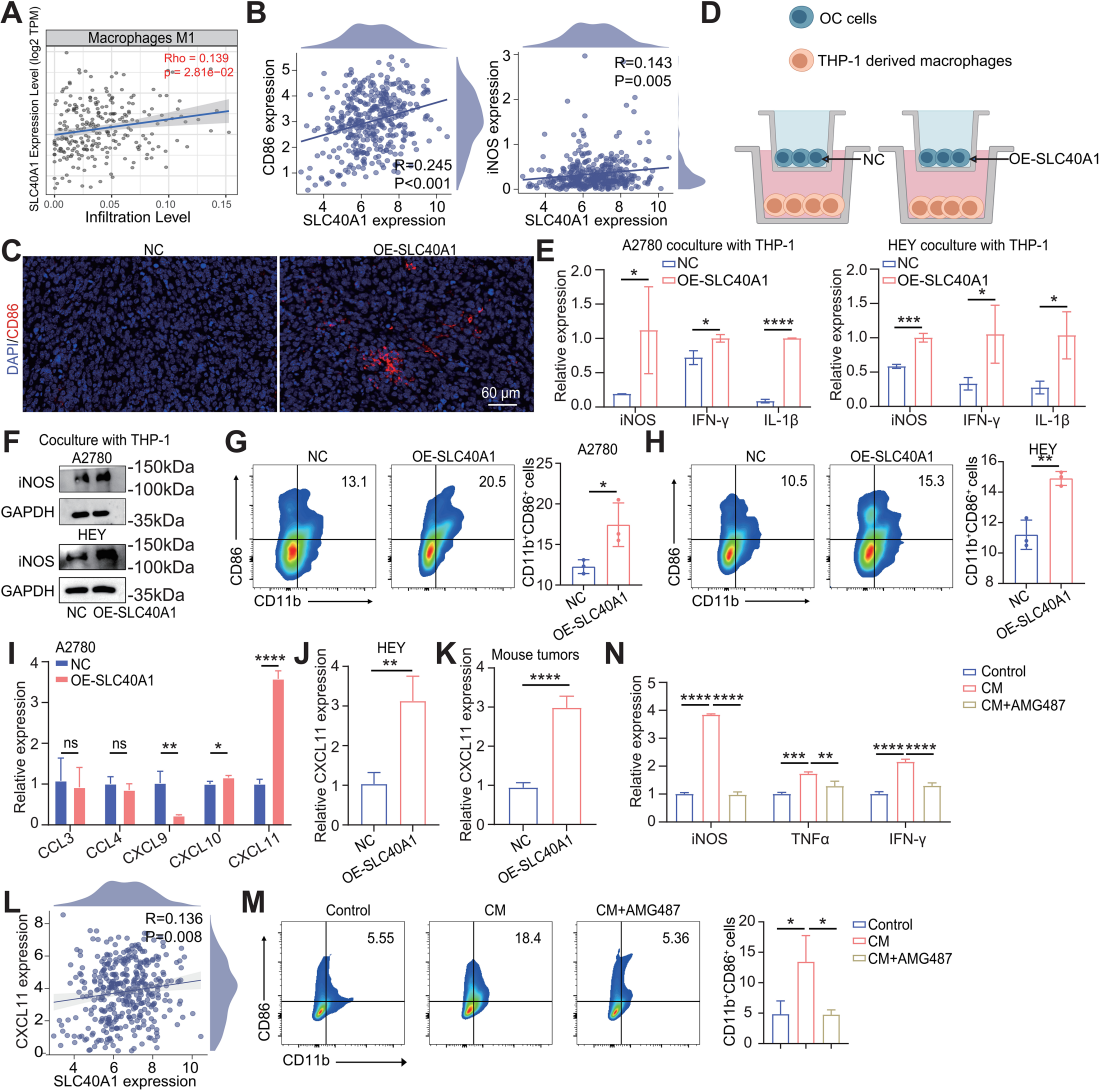
SLC40A1 drives M1 macrophage polarization via the CXCL11–CXCR3 axis in epithelial ovarian cancer (EOC). **(A)** Correlation analysis between SLC40A1 and M1 macrophage infiltration based on TIMER database (R = 0.139; p = 0.0281). **(B)** Correlation analysis between SLC40A1 and CD86 (left) and iNOS (right) expression based on TCGA-OV cohort. **(C)** Representative immunofluorescence (IF) images showing CD86 expression in orthotopic ovarian tumor models [negative control (NC) and OE-SLC40A1 groups; n = 3]. Scale bar, 60 μm. **(D)** Simple model of co-culture of tumor cells and macrophages; macrophages (placed in the lower chamber of co-culture) were co-cultured with EOC cell lines (A2780, HEY) with or without OE-SLC40A1 for 48 h. **(E)** qPCR analysis of iNOS, IFN-γ, and IL-1β mRNA expression in macrophages co-cultured with A2780 (left) or HEY (right) cells (NC and OE-SLC40A1 groups). **(F)** Western blotting (WB) analysis of iNOS and protein levels in macrophages co-cultured with A2780 (up) or HEY (down) cells under NC or SLC40A1-overexpressing conditions. **(G, H)** Flow cytometry analysis showing CD86 expression in macrophages after co-culture with A2780 **(G)** or HEY **(H)** cells with NC or SLC40A1 overexpression. **(I)** qPCR analysis of CCL3, CCL4, CXCL9, CXCL10, and CXCL11 mRNA expression in A2780 cells (NC and OE-SLC40A1 groups). **(J)** qPCR analysis of CXCL11 mRNA expression in HEY cells (NC and OE-SLC40A1 groups). **(K)** qPCR analysis of CXCL11 mRNA expression in ovarian tumor models between two groups (n = 4). Data are presented as the means ± SD. **(L)** Correlation analysis between SLC40A1 and CXCL11 expression based on TCGA-OV cohort. **(M)** The proportion of CD11b^+^CD86^+^ macrophages was assessed in control, conditioned medium (CM) from SLC40A1-overexpressing A2780 cell-treated, and CM+AMG487-treated groups (left), and quantitative analysis was performed (right). **(N)** qPCR analysis was performed to examine the mRNA expression levels of iNOS, TNF-α, and IFN-γ in the control, CM-treated, and CM+AMG487-treated macrophage groups. Unless otherwise noted, data are presented as the means ± SD (n = 3). Statistical analysis was performed using unpaired two-sided Student’s t-test **(E, G–K)** and one-way ANOVA followed by Dunnett’s multiple comparisons test **(M, N)**. *p < 0.05, **p < 0.01, ***p < 0.001, and ****p < 0.0001.

Building upon the observation that SLC40A1 promotes macrophage infiltration into the TME, we next sought to determine whether SLC40A1 also influences macrophage polarization. Macrophages within tumors exhibit remarkable plasticity and are broadly categorized into classically activated M1-like macrophages, which exert antitumor activity through pro-inflammatory and immune-stimulatory functions, and alternatively activated M2-like macrophages, which typically support tumor progression (22, 24). Given the prognostic implications of SLC40A1 in relation to macrophage infiltration, we hypothesized that SLC40A1 may preferentially modulate the polarization state of macrophages, particularly driving an M1-like phenotype to enhance antitumor immunity. By querying the TIMER database, we observed that SLC40A1 expression levels were significantly and positively correlated with M1 macrophage infiltration in OC (**Figure 4A**). Moreover, SLC40A1 expression was also positively associated with the expression of canonical M1 markers, including CD86 and iNOS (**Figure 4B**). These findings were further validated in tumor tissues from the mouse tumor model, in which tumors derived from SLC40A1-overexpressing cells exhibited higher CD86 expression compared with the control group (**Figure 4C**).

To further confirm the association between tumor-derived SLC40A1 and M1 macrophage polarization in the TME, we employed a Transwell co-culture system, in which tumor cells were placed in the upper chamber and macrophages in the lower chamber, allowing paracrine interactions without direct cell–cell contact (**Figure 4D**). The co-culture results revealed that high SLC40A1 expression in tumor cells led to increased mRNA levels of M1 macrophage markers, including iNOS, IFN-γ, and IL-1β, whereas the expression of M2-associated markers, such as CD163, IL-10, and TGFβ, remained largely unchanged (**Figure 4E**, **Supplementary Figure S3A**). Consistently, WB analysis confirmed the upregulation of iNOS protein, and flow cytometry further demonstrated increased expression of the M1 surface marker CD86 (**Figures 4F–H**). As anticipated, flow cytometry further demonstrated no significant change in the M2 surface marker CD206 (**Supplementary Figures S3B, C**).

Since SLC40A1 overexpression in tumor cells selectively promoted M1 but not M2 macrophage polarization, we reasoned that tumor-derived soluble factors may mediate this effect. Considering that chemokines are key regulators of immune cell recruitment and polarization within the TME, we subsequently focused on whether tumor cell-secreted chemokines contribute to the induction of M1 macrophages (25, 26). CCL3, CCL4, CXCL9, CXCL10, and CXCL11 are known to facilitate M1 macrophage polarization; therefore, we next analyzed their expression patterns in tumor cells (27–29). qPCR analysis confirmed that among the examined chemokines, CXCL11 exhibited the most pronounced upregulation in tumor cells with elevated SLC40A1 expression (**Figures 4I, J**). This finding was further corroborated via ELISA, which also demonstrated a significant elevation of CXCL11 in SLC40A1-overexpressing tumor cells (**Supplementary Figure S4**). Evidence from murine tumor tissues, together with TCGA expression datasets, collectively supports a positive regulatory relationship between SLC40A1 and CXCL11 in OC (**Figures 4K, L**). Notably, CXCL11 exerts its effects mainly via the high-affinity receptor CXCR3, expressed on activated CD8^+^ T cells, NK cells, and macrophages, suggesting that the SLC40A1–CXCL11 axis may facilitate M1 macrophage polarization through CXCR3 signaling (30, 31). To validate this possibility, we first treated macrophages with recombinant CXCL11 protein, followed by exposure to AMG487, a selective antagonist of the chemokine receptor CXCR3. We found that CXCL11 promoted macrophage polarization toward the M1 phenotype, whereas AMG487 partially reversed this effect. These results were supported by flow cytometry analysis of CD86 expression and qPCR measurements of iNOS, IFN-γ, and TNF-α (**Figures 4M, N**). In summary, SLC40A1 promotes M1 macrophage polarization in EOC via the CXCL11–CXCR3 axis.

### 3.5 SLC40A1–CXCL11 signaling activates the JAK2–STAT1 pathway and establishes a positive feedback loop in EOC

**Figure 5:**
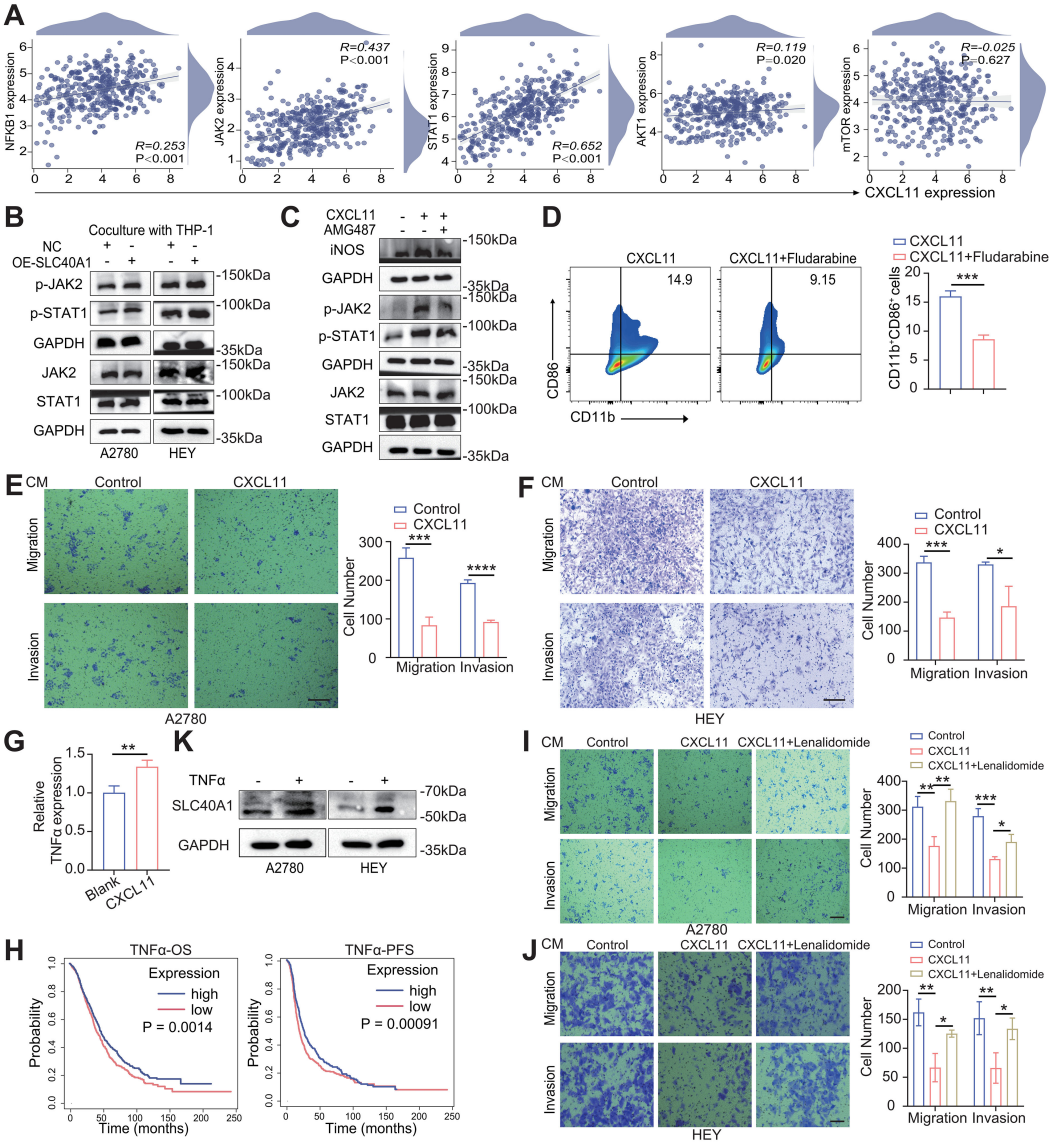
SLC40A1–CXCL11 axis promotes M1 polarization via JAK2–STAT1 activation and forms a positive feedback loop in epithelial ovarian cancer (EOC). **(A)** Correlation analysis between CXCL11 and NFKB1, JAK2, STAT1, AKT1, and mTOR expression based on TCGA-OV cohort. **(B)** Western blotting (WB) analysis was performed to examine the total JAK2, STAT1, and p-JAK2, p-STAT1 in macrophages co-cultured with control or SLC40A1-overexpressing tumor cells, with A2780 on the left and HEY on the right. Representative WB images are displayed. **(C)** WB showing the expression of JAK2, p-JAK2, STAT1, p-STAT1, and iNOS in macrophages under three conditions: control, CXCL11 treatment, and CXCL11 plus AMG487 treatment. **(D)** Flow cytometry analysis of CD11b^+^CD86^+^ macrophages in CXCL11-treated and CXCL11+fludarabine-treated groups (left), with corresponding quantitative results shown on the right. **(E, F)** Transwell assays were performed to evaluate the migration and invasion of A2780 **(E)** and HEY **(F)** cells treated with conditioned medium (CM) from macrophages under the indicated conditions. Scale bar, 100 μm. Representative images of migrated cells are shown at the top, while representative images of invaded cells are shown at the bottom. **(G)** qPCR quantification of TNF-α expression in macrophages comparing untreated controls and CXCL11-treated cells. **(H)** The TCGA-OV datasets were analyzed to examine the effects of TNF-α levels on overall survival (OS) and progression-free survival (PFS) of patients. Statistical analysis was performed using the log-rank test. **(I, J)** Migration and invasion of A2780 **(I)** and HEY **(J)** cells were evaluated following treatment with CM from macrophages under the indicated conditions, with representative images of migrated cells on the top and invaded cells on the bottom. Scale bar, 100 μm. **(K)** Expression of SLC40A1 in A2780 (left) and HEY (right) cells after TNF-α treatment, as determined by WB. Data are presented as the means ± SD [**(D–G, I, J)**; n = 3]. Statistical analysis was performed using unpaired two-sided Student’s t-test **(D–G)** and one-way ANOVA followed by Dunnett’s multiple comparisons test **(I, J)**. *p < 0.05, **p < 0.01, ***p < 0.001, and ****p < 0.0001.

Macrophage polarization is often accompanied by the activation of intracellular signaling cascades, among which NF-κB, JAK2–STAT1, and AKT–mTOR pathways are typically implicated in M1 macrophage activation (28, 32–34). To further elucidate the mechanism underlying SLC40A1-induced M1 polarization, we next examined whether these signaling pathways were involved. Therefore, we initially utilized the TCGA-OV cohort to assess the correlation between CXCL11 expression and key molecules involved in major signaling pathways, including NFKB1, JAK2, STAT1, AKT1, and mTOR. The analysis revealed that CXCL11 exhibited a significantly stronger association with JAK2–STAT1 molecules compared with the other pathways (**Figure 5A**). We then performed WB analysis to examine the activation of the JAK2–STAT1 signaling pathway in macrophages co-cultured with control or SLC40A1-overexpressing tumor cells. The results showed that macrophages co-cultured with SLC40A1-overexpressing tumor cells exhibited enhanced phosphorylation of JAK2 and STAT1 (p-JAK2 and p-STAT1, respectively) (**Figure 5B**). Considering the regulatory role of SLC40A1 on CXCL11 expression and M1 macrophage polarization, we hypothesized that CXCL11 itself may activate the JAK2–STAT1 pathway to drive M1 polarization (35). To test this, we treated macrophages with recombinant CXCL11 protein, and we observed that CXCL11 treatment similarly enhanced p-JAK2 and p-STAT1. Moreover, CXCL11-treated macrophages exhibited increased expression of iNOS (**Figure 5C**). Importantly, the addition of AMG487 partially reversed these effects, and the inhibition of p-STAT1 by fludarabine also abrogated CXCL11-induced M1 polarization, as evidenced by reduced CD86 expression (**Figures 5C, D**), indicating that CXCL11 is sufficient to promote M1 polarization via JAK2–STAT1 activation.

Next, we sought to investigate the functional consequences of SLC40A1-induced macrophage reprogramming on tumor cells. Given that SLC40A1 upregulates CXCL11 expression, and considering that macrophages exert their regulatory effects not only through direct cell–cell interactions but also via the secretion of soluble mediators, we examined whether the CM derived from SLC40A1-induced macrophages could influence the biological behavior of EOC cells (36–38) (**Supplementary Figure S5A**). These findings suggest that CXCL11 acts as a critical mediator through which macrophages exert their antitumor effects. Specifically, the CM from CXCL11-treated macrophages markedly suppressed the migration and invasion of EOC cells, indicating that CXCL11 contributes to restraining malignant behaviors (**Figures 5E, F**). In line with this functional evidence, survival analysis further demonstrated that elevated CXCL11 expression was significantly associated with improved prognosis in patients with OC (**Supplementary Figure S5B**). qPCR analysis confirmed that CXCL11-treated macrophages exhibited increased TNF-α expression (**Figure 5G**). Given its established role as a key M1-associated cytokine with antitumor activity and its positive correlation with patient prognosis (**Figure 5H**), we next disrupted TNF-α in the CM using the TNF-α inhibitor lenalidomide (39), which effectively reversed the inhibitory effects on EOC cell migration and invasion (**Figures 5I,J**). Interestingly, we further observed that the treatment of EOC cells with TNF-α led to the upregulation of SLC40A1 (**Figure 5K**), highlighting a reciprocal regulatory loop in which TNF-α produced by CXCL11-stimulated macrophages induces SLC40A1 expression in EOC cells, thereby potentially sustaining M1 polarization and antitumor activity.

### 3.6 SLC40A1 expression correlates with the TIME and immunotherapy response in EOC

**Figure 6:**
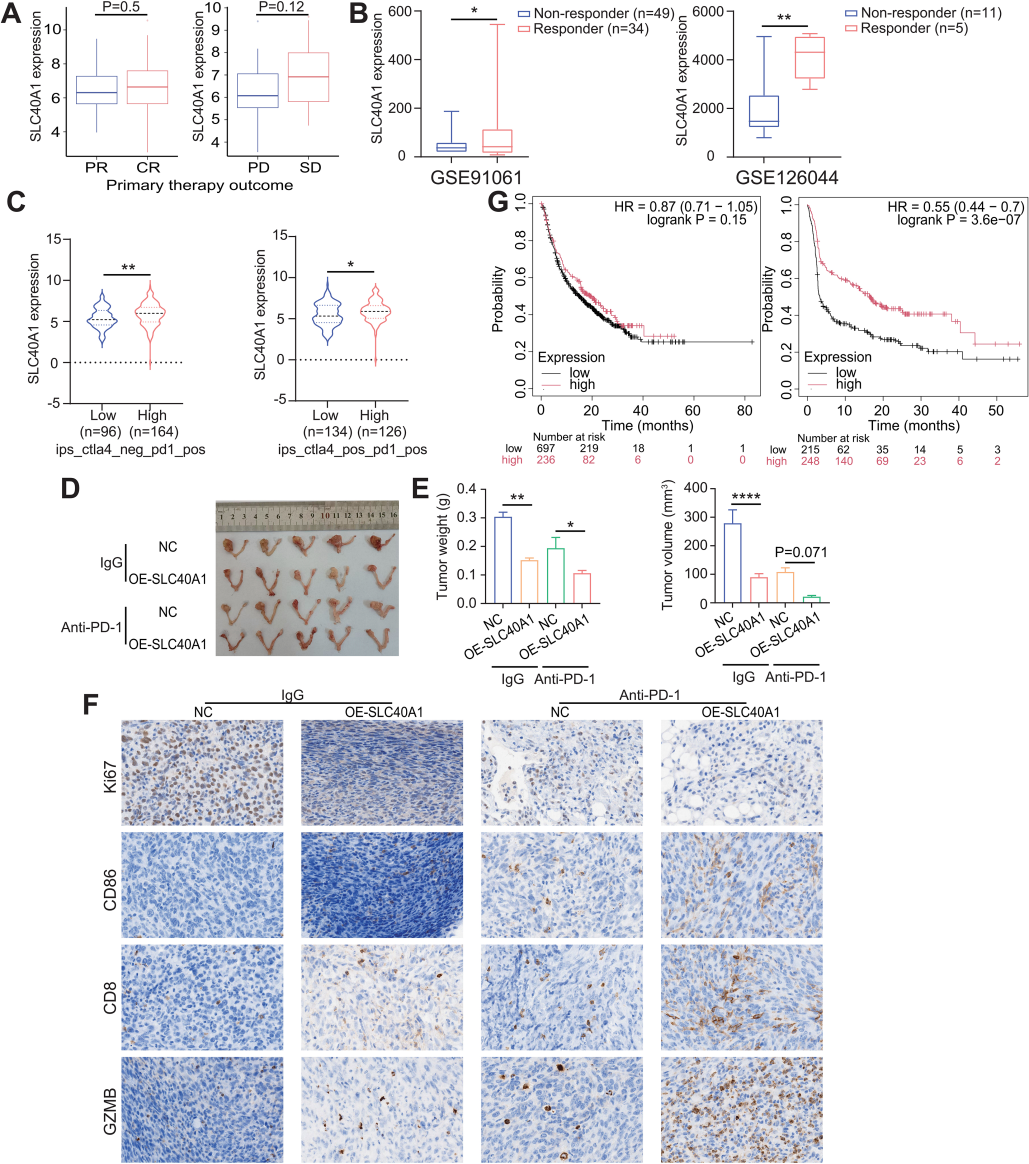
SLC40A1 links the tumor immune microenvironment (TIME) to therapeutic response in epithelial ovarian cancer (EOC). **(A)** Expression level of SLC40A1 in different subgroups [complete responders (CRs) and partial responders (PRs); progressive disease (PD) and stable disease (SD)] of prime therapy-treated The Cancer Genome Atlas (TCGA) clinical cohort. **(B)** Analysis of SLC40A1 expression levels in different subgroups of immunotherapy-treated clinical cohorts comparing non-responders and responders in GSE91061 (left) and GSE126044 (right). **(C)** Based on different immunophenoscores (IPSs) of CTLA4_neg PD1_pos (left) and CTLA4_pos PD1_pos (right), the expression of SLC40A1 was compared between groups. **(D)** The images of orthotopic tumors of the ID8 cells in C57BL/6 mice (n = 5 mice per group) are shown. **(E)** The weight (left) and volume (right) of tumor tissues in C57BL/6 mice between groups (n = 5 mice per group) were compared. **(F)** The Ki67, CD86, CD8, and GZMB were evaluated by immunohistochemistry (IHC) in tumor tissues of tumor-bearing mice (n = 3). All panels are of the same magnification. Scale bar, 100 μm. **(G)** Comparison of overall survival (OS) (left) and progression-free survival (PFS) (right) in the combined cancer types (bladder, glioblastoma, melanoma, and other tumors) patients who had high SLC40A1 versus low SLC40A1 intra‐tumoral expression receiving anti‐PD‐1. Survival analysis was analyzed using log‐rank test. Data are presented as the means ± SD **(A)** or means ± SEM **(E)**. Data are presented as values ranging from minimum to maximum **(B, C)**. Statistical analysis was performed using unpaired two-sided Student’s t-test **(A–C)** and one-way ANOVA followed by Tukey’s multiple comparisons test **(E)**. *p < 0.05, **p < 0.01, and ***p < 0.001.

Considering that SLC40A1 influences macrophage-mediated antitumor immunity through the CXCL11–TNF-α axis, we next aimed to investigate its potential impact on the TIME and its relevance to the efficacy of immune checkpoint therapies in OC. An initial analysis of the TCGA-OV cohort demonstrated that SLC40A1 expression was elevated in complete responders (CRs) relative to partial responders (PRs), and in patients with stable disease (SD) compared with those with progressive disease (PD), implicating SLC40A1 in therapeutic sensitivity (**Figure 6A**). Furthermore, analysis of immunotherapy-treated cohorts (GSE91061 and GSE126044) revealed higher SLC40A1 expression in responders than in non-responders (**Figure 6B**). More importantly, the interrogation of The Cancer Immunome Atlas (https://tcia.at/home) database showed that SLC40A1 expression was markedly higher in groups with elevated immunophenoscore (IPS) under both CTLA4_neg PD1_pos and CTLA4_pos PD1_pos settings (**Figure 6C**).

**Figure 7:**
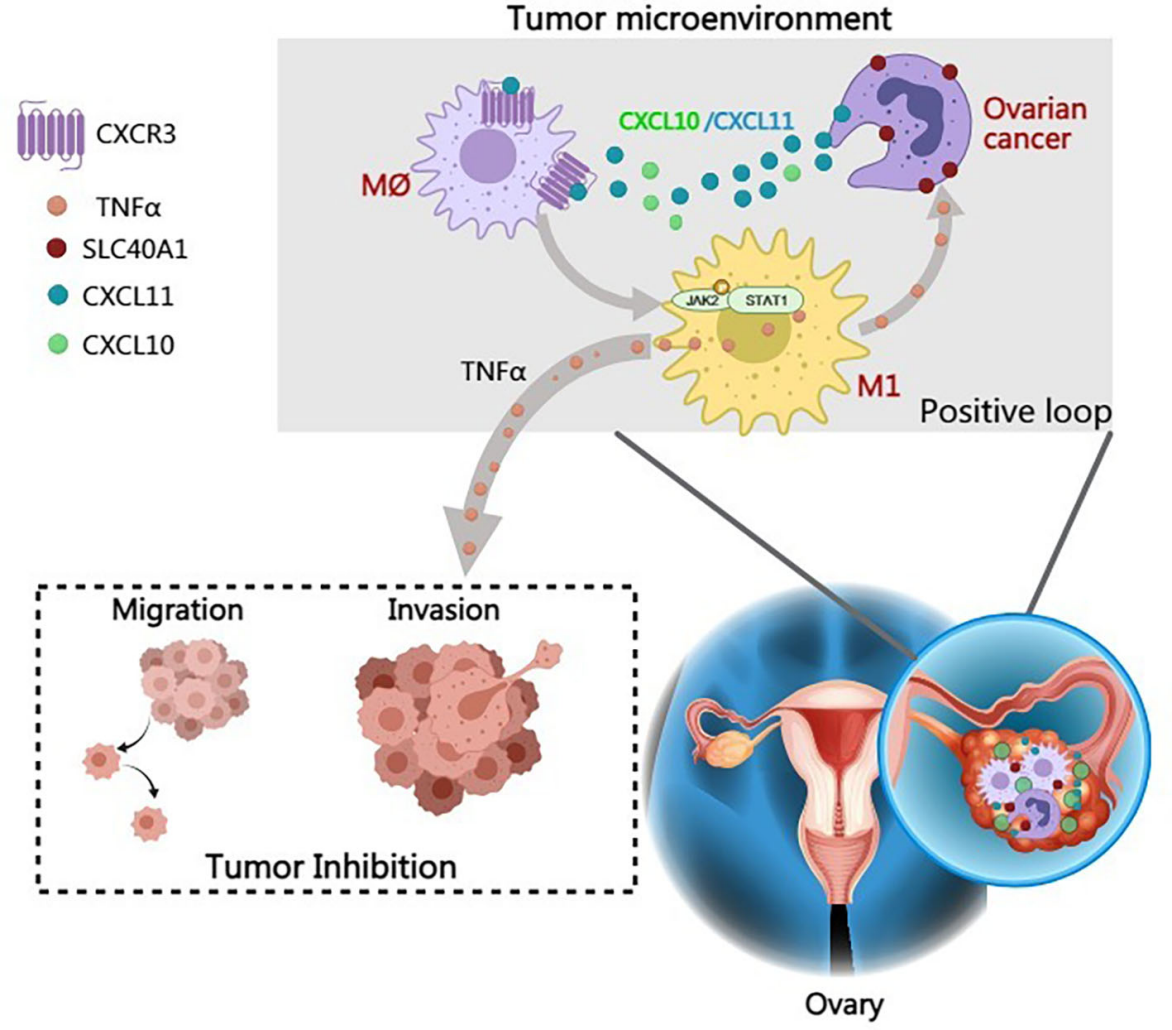
Graphic abstract of SLC40A1 in modulating the tumor immune microenvironment (TIME) in epithelial ovarian cancer (EOC). SLC40A1 enhances macrophage polarization toward the M1 phenotype through activation of the JAK2–STAT1 signaling pathway. Activated M1 macrophages secrete TNF-α, which in turn promotes the overexpression of SLC40A1 in tumor cells. This establishes a positive feedback loop that reinforces M1 macrophage activation, amplifies pro-inflammatory immune responses, and ultimately suppresses ovarian cancer (OC) progression while improving responsiveness to immunotherapy.

Given the enrichment of SLC40A1 in IPS-high groups under PD-1 blockade settings, we next sought to experimentally validate its functional role *in vivo* and to assess whether SLC40A1 overexpression could potentiate the efficacy of anti-PD-1 therapy. We established an orthotopic ID8 allograft mouse model and treated tumor-bearing mice with anti-PD-1. While either SLC40A1 overexpression or PD-1 blockade alone inhibited tumor growth, their combination produced the most pronounced antitumor effect. Specifically, anti-PD-1 monotherapy exerted therapeutic activity, but the addition of SLC40A1 overexpression synergistically enhanced its efficacy, leading to markedly reduced tumor growth (**Figures 6D, E**). Moreover, IHC staining of tumor tissues revealed decreased Ki67 expression, confirming reduced proliferative activity. In addition, we observed increased expression of CD86, as well as enhanced infiltration of CD8^+^ T cells and elevated levels of GZMB, further supporting the role of SLC40A1 in promoting antitumor immunity (**Figure 6F**). To extend the clinical relevance of these findings, we observed that patients with high SLC40A1 expression who received anti‐PD‐1 therapy exhibited improved survival compared to those with low SLC40A1 expression (**Figure 6G**). Collectively, these findings indicate that high SLC40A1 expression is associated with favorable therapeutic responses and enhanced sensitivity to immune checkpoint blockade. Additionally, **Figure 7** presents a comprehensive and systematic overview of the pathways, processes, and mechanisms explored in this study.

## 4 Discussion

EOC remains one of the most aggressive gynecological malignancies, with high mortality largely due to late-stage diagnosis, frequent recurrence, and limited response to conventional therapies (40). Despite advances in surgery, chemotherapy, and emerging immunotherapies, the prognosis for advanced EOC patients remains poor, highlighting the urgent need for novel molecular targets and strategies that modulate the TIME (41).

In this study, we demonstrated that SLC40A1, a key regulator of iron metabolism, is downregulated in EOC tissues compared to normal ovarian tissues, and its high expression correlates with improved patient prognosis. Interestingly, while SLC40A1 did not directly influence tumor cell proliferation, apoptosis, or migration *in vitro*, our *in vivo* experiments revealed that it exerts profound immunoregulatory effects, particularly through the modulation of macrophage polarization. We found that SLC40A1 promotes M1 macrophage polarization, thereby enhancing macrophage-mediated antitumor activity. This is consistent with growing evidence that M1 macrophages contribute to tumor suppression by producing pro-inflammatory cytokines and activating adaptive immune responses, whereas dysregulated macrophage polarization can facilitate tumor progression (42–45).

To support these mechanistic studies, we used HEY and A2780 human OC cell lines for *in vitro* experiments and ID8 murine cells for *in vivo* orthotopic modeling. HEY and A2780 provide complementary molecular backgrounds, and their relatively low endogenous SLC40A1 expression makes them suitable for overexpression studies, while ID8 allows the establishment of syngeneic tumors in immunocompetent mice to assess tumor–immune interactions. We acknowledge that these models do not cover all EOC subtypes and that ID8 is a murine line, which may not fully recapitulate human disease. Nevertheless, the combination of these *in vitro* and *in vivo* models offers a robust platform for investigating SLC40A1-mediated immunoregulatory mechanisms. Future studies using additional human cell lines and patient-derived xenograft models will help further validate and extend these findings.

Mechanistically, our findings uncovered a positive feedback loop in which SLC40A1 regulates CXCL11 secretion, activating the JAK2–STAT1 pathway in macrophages and inducing TNF-α production, which in turn further upregulates SLC40A1 expression. This loop not only reinforces M1 polarization but also establishes a self-sustaining antitumor immune environment. Such a mechanism highlights a previously underappreciated link between SLC40A1 and immune regulation in EOC. Importantly, we demonstrated that SLC40A1 enhances the response to immunotherapy, suggesting that the modulation of the SLC40A1–M1 macrophage axis could improve therapeutic outcomes. These findings provide a rationale for considering SLC40A1 as both a prognostic biomarker and a potential target for immunotherapeutic intervention.

Furthermore, given that the role of SLC40A1 in tumor immunity has been largely unexplored, our study offers novel insights into how metabolic regulators can shape the TIME. Although our study demonstrates that SLC40A1 regulates the CXCL11–JAK2–STAT1 axis to promote M1 macrophage polarization in EOC, we acknowledge that the current work does not fully explore all unique aspects of how this signaling axis operates within the ovarian TME. Additional tumor- and microenvironment-specific factors may influence this pathway, and further investigations are required to elucidate these contributions. Nonetheless, our study provides novel mechanistic insight by identifying SLC40A1 as an upstream regulator linking tumor cells to immune modulation, emphasizing its potential impact on shaping the TIME and informing therapeutic strategies.

Future studies are warranted to explore the translational potential of SLC40A1-targeted strategies in clinical settings, including their interaction with other immune cell populations and their impact on combination therapies. Additionally, further mechanistic investigations are needed to determine how SLC40A1 expression is regulated in EOC and whether its modulation can synergize with existing immunotherapies to achieve durable antitumor responses.

In summary, our work establishes SLC40A1 as a critical modulator of macrophage-mediated antitumor immunity in EOC and highlights the therapeutic potential of targeting the SLC40A1–M1 macrophage axis to suppress tumor progression and enhance immunotherapy efficacy.

## Data Availability

The raw data supporting the conclusions of this article will be made available by the authors, without undue reservation.
