# Supplementary material for: SLC40A1-mediated positive feedback loop with M1 macrophages suppresses epithelial ovarian cancer progression
Source: Front Immunol. 2026 Jan 14;16:1709597. doi: 10.3389/fimmu.2025.1709597 (PMC12847247; doi:10.3389/fimmu.2025.1709597)
Supplement: Supplementary file 1 [file DataSheet1.docx]

**SLC40A1-mediated positive feedback with M1 macrophages suppresses epithelial ovarian cancer progression**

The Supplementary Material for this article includes the

**original western blotting gels,**

**Supplementary Tables 1–3,**

**Supplementary Figures 1–5**

**Original western blot gels,**

**
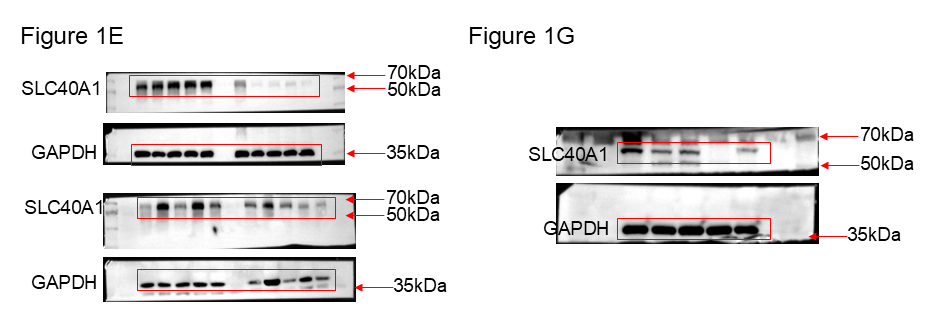
**

**
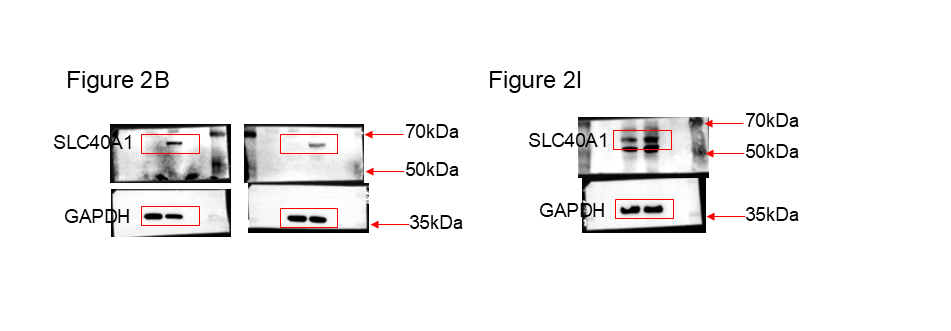
**

**
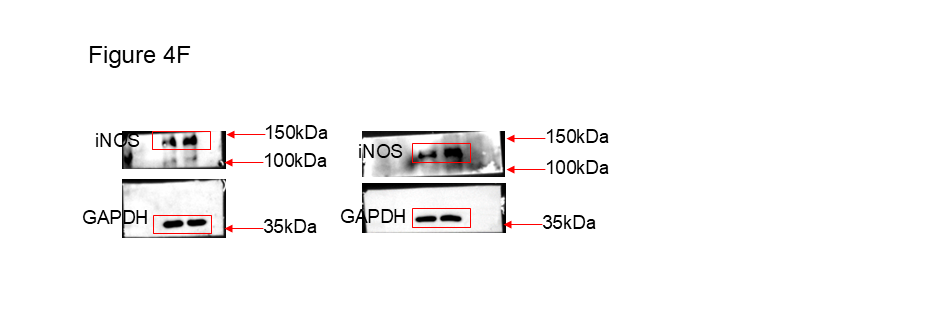

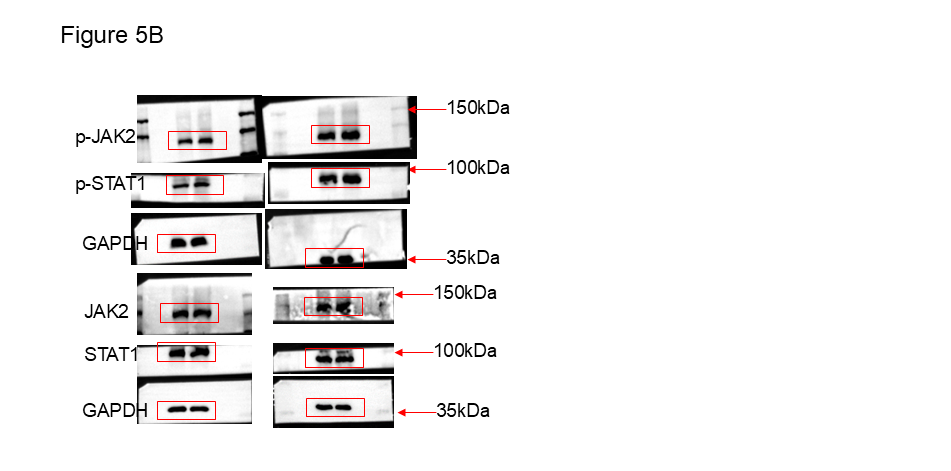
**

**
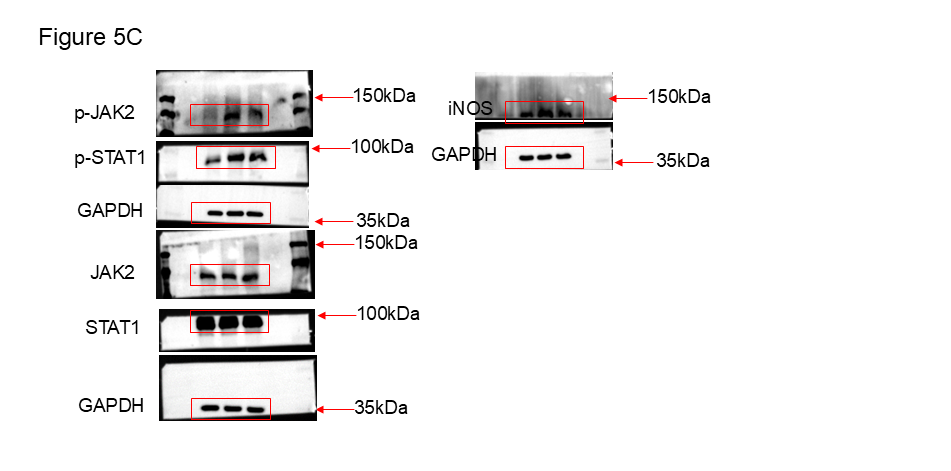
**

**
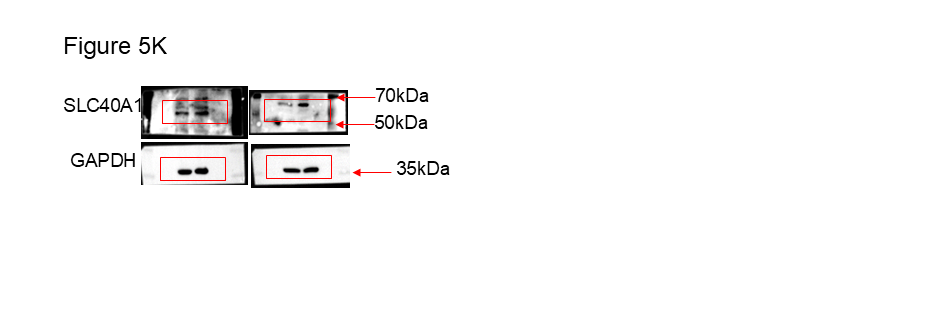
**

**Supplementary Table S1** Flow cytometry antibodies and reagents

| **Antibodies** | **Source** | **Identifier** |
| --- | --- | --- |
| Zombie Aqua™ Fixable Viability Kit | Biolegend | Cat#423101 |
| Brilliant Violet 421™ anti-mouse CD45 | Biolegend | Cat#103134 |
| PerCP/Cyanine5.5 anti-mouse F4/80 | Biolegend | Cat#123128 |
| APC anti-mouse/human CD11b | Biolegend | Cat#101212 |
| PE Anti-Human CD86 Antibody | Elabscience Biotechnology | Cat#E-AB-F1012D |
| FITC anti-human CD11b | Biolegend | Cat#982614 |
| APC Anti-Human CD206/MMR Antibody | Elabscience Biotechnology | Cat#E-AB-F1161E |

| **Reagents** | **Source** | **Identifier** |
| --- | --- | --- |
| Immobilon® -P PVDF Membrane | SigmaAldrich | IPVH00010 |
| Nonfat milk | Epizyme | PS112 |
| Tris - Buffered Saline with Tween (TBST) | Epizyme | PS103 |
| InVivoMAb anti-mouse PD-1 (CD279) | STARTER | Cat#S0B0594 |
| AMG 487 | MedChemExpress | Cat# HY-15319 |
| Phorbol 12-myristate 13-acetate | MedChemExpress | Cat#HY-18739 |
| I-TAC/CXCL11 Protein, Human | MedChemExpress | Cat#HY-P7229 |
| Lenalidomide | MedChemExpress | Cat#HY-A0003 |
| Fludarabine | MedChemExpress | Cat#HY-B0069 |
| TNF alpha/TNFSF2 Protein, Human | MedChemExpress | Cat#HY-P70426A |

**Supplementary Table S2 qPCR primer list**

| Gene name | Species | Forward Sequence | Reverse Sequence |
| --- | --- | --- | --- |
| GAPDH | Human | AAAGGCATTCTTCACCTGCTCC | GCCATCACGCCACAGTTTC |
| SLC40A1 | Human | CTACTTGGGGAGATCGGATGT | CTGGGCCACTTTAAGTCTAGC |
| CXCL10 | Human | GTGGCATTCAAGGAGTACCTC | TGATGGCCTTCGATTCTGGATT |
| CXCL11 | Human | GACGCTGTCTTTGCATAGGC | GGATTTAGGCATCGTTGTCCTTT |
| CXCL11 | Mouse | TGTAATTTACCCGAGTAACGGC | CACCTTTGTCGTTTATGAGCCTT |
| F4/80 (Adgre1) | Mouse | CTCAGTCTGCACCAATATCCTG | CCACAGAGTTAGAGCAGTTGGAA |
| β-actin | Mouse | GTGACGTTGACATCCGTAAAGA | GCCGGACTCATCGTACTCC |
| iNOS (NOS2) | Human | TTCAGTATCACAACCTCAGCAAG | TGGACCTGCAAGTTAAAATCCC |
| IFN-γ (IFNG) | Human | TCGGTAACTGACTTGAATGTCCA | TCGCTTCCCTGTTTTAGCTGC |
| IL-1β (IL1B) | Human | ATGATGGCTTATTACAGTGGCAA | GTCGGAGATTCGTAGCTGGA |
| CCL3 | Human | AGTTCTCTGCATCACTTGCTG | CGGCTTCGCTTGGTTAGGAA |
| CCL4 | Human | CTGTGCTGATCCCAGTGAATC | TCAGTTCAGTTCCAGGTCATACA |
| CXCL9 | Human | CCAGTAGTGAGAAAGGGTCGC | AGGGCTTGGGGCAAATTGTT |
| TNFα (TNF) | Human | CCTCTCTCTAATCAGCCCTCTG | GAGGACCTGGGAGTAGATGAG |

**Supplementary Table S3 TCGA Cancer Abbreviations and Full Names**

| Abbreviations | Full titles |
| --- | --- |
| BRCA | Breast Invasive Carcinoma |
| CESC | Cervical Squamous Cell Carcinoma and Endocervical Adenocarcinoma |
| ESCA | Esophageal carcinoma |
| HNSC | Head and Neck Squamous Cell Carcinoma |
| KIRP | Kidney renal papillary cell carcinoma |
| LIHC | Liver hepatocellular carcinoma |
| OV | Ovarian Serous Cystadenocarcinoma |
| READ | Rectum Adenocarcinoma |
| PAAD | Pancreatic adenocarcinoma |

**Supplementary Figure S1-S6**

**Supplementary Figure S1**

**
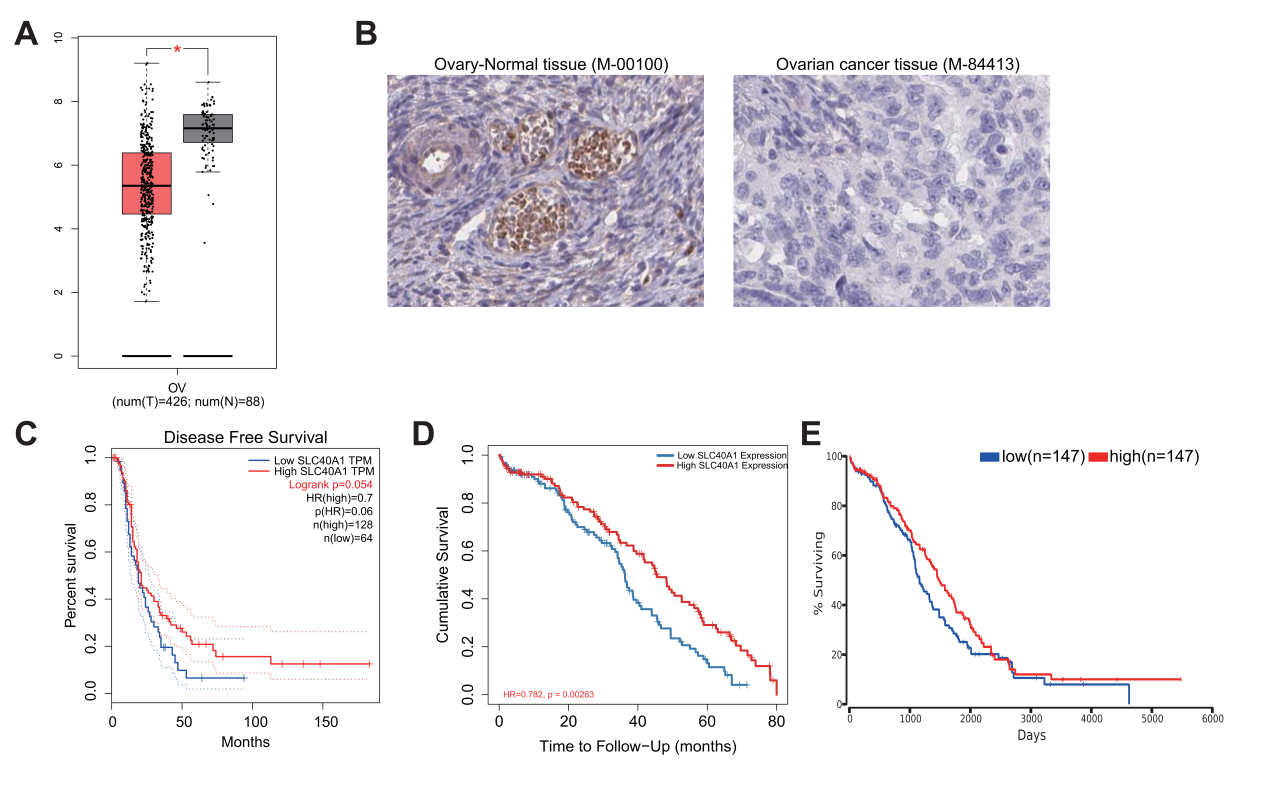
**

**Fig. S1 Expression and clinical significance of SLC40A1 in EOC.**

(A-B) Further validation of SLC40A1 overexpression in ovary normal tissues through GEPIA (A) (gepia.cancer-pku.cn) and HPA (B) (https://www.proteinatlas.org/) databases.

(C) Further validation supported the association between high SLC40A1 expression and a favorable prognosis through GEPIA (C), TIMER2.0 (D) (timer.cistrome.org) and OncoLnc (E) (www.OncoLnc.org). Statistical analysis was performed using the log-rank test.

**Supplementary Figure S2**


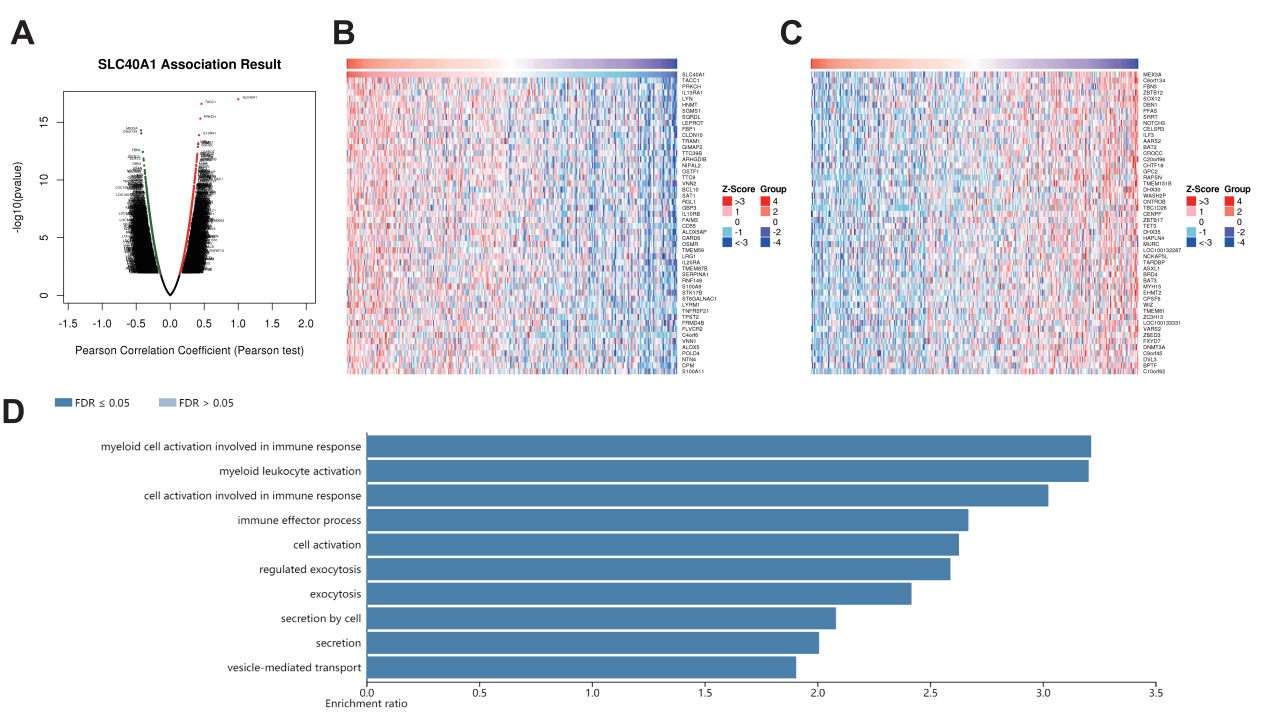


**Figure S2 Enrichment analysis of expression profile characteristics of SLC40A1 in EOC.**

(A-C) Analyses were performed using The LinkedOmics (http://www.linkedomics.org/) database. (A) The volcano plot illustrates the correlation of co-expressed genes with SLC40A1 in EOC. (B-C) The heatmap displays the top 50 genes in EOC that exhibit the strongest positive (left) and negative (right) correlations with SLC40A1. (D) Gene Ontology enrichment analyses of SLC40A1 co-expressed genes.

**Supplementary** **Figure S3**


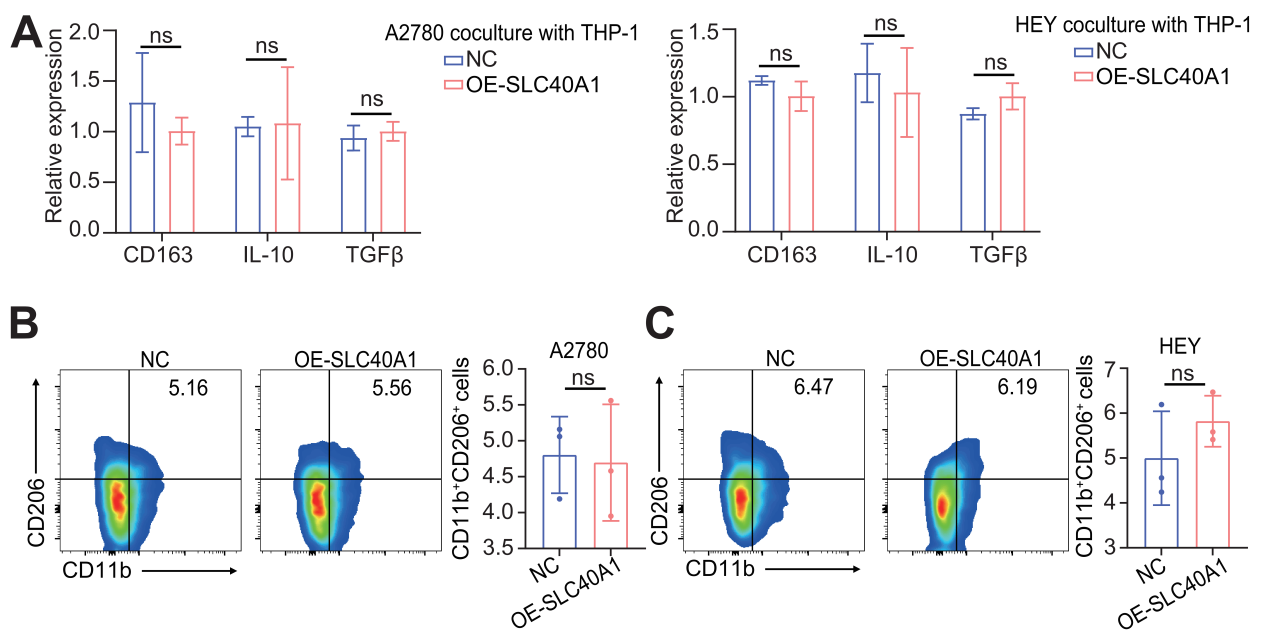


**Figure S3 Changes in SLC40A1 expression in EOC do not influence M2 macrophage polarization.**

(A) qPCR analysis of CD163, IL-10, and TGFβ mRNA expression in macrophages co-cultured with A2780 (left) or HEY (right) cells (NC and OE-SLC40A1 groups).

(B-C) FCM analysis showing the proportion of CD11b^+^CD206^+^ macrophages after co-culture with A2780 (B) or HEY (C) cells with NC or SLC40A1 overexpression.

Data are presented as the means ± SD (n = 3). Statistical analysis was performed by unpaired two-sided Student’s t-test. ns, not significant.

**Supplementary Figure S4**


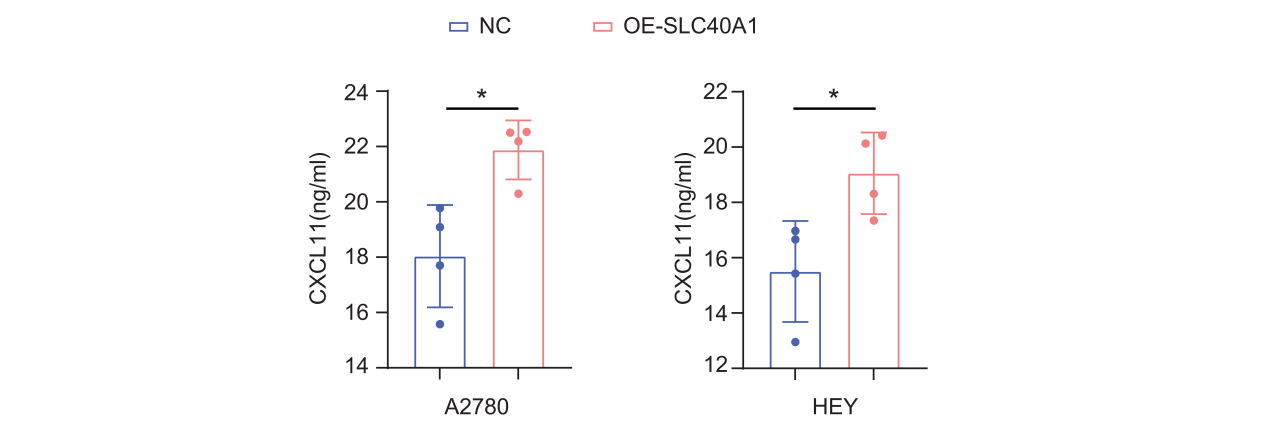


**Figure S4 SLC40A1 levels are positively correlated with CXCL11 in EOC.**

The levels of CXCL11 in the supernatants of control and SLC40A1-overexpressing EOC cell lines A2780 (left) and HEY (right) were measured by ELISA. Data are presented as the means ± SD (n = 4). Statistical analysis was performed by unpaired two-sided Student’s t-test. *p<0.05.

**Supplementary Figure S5**


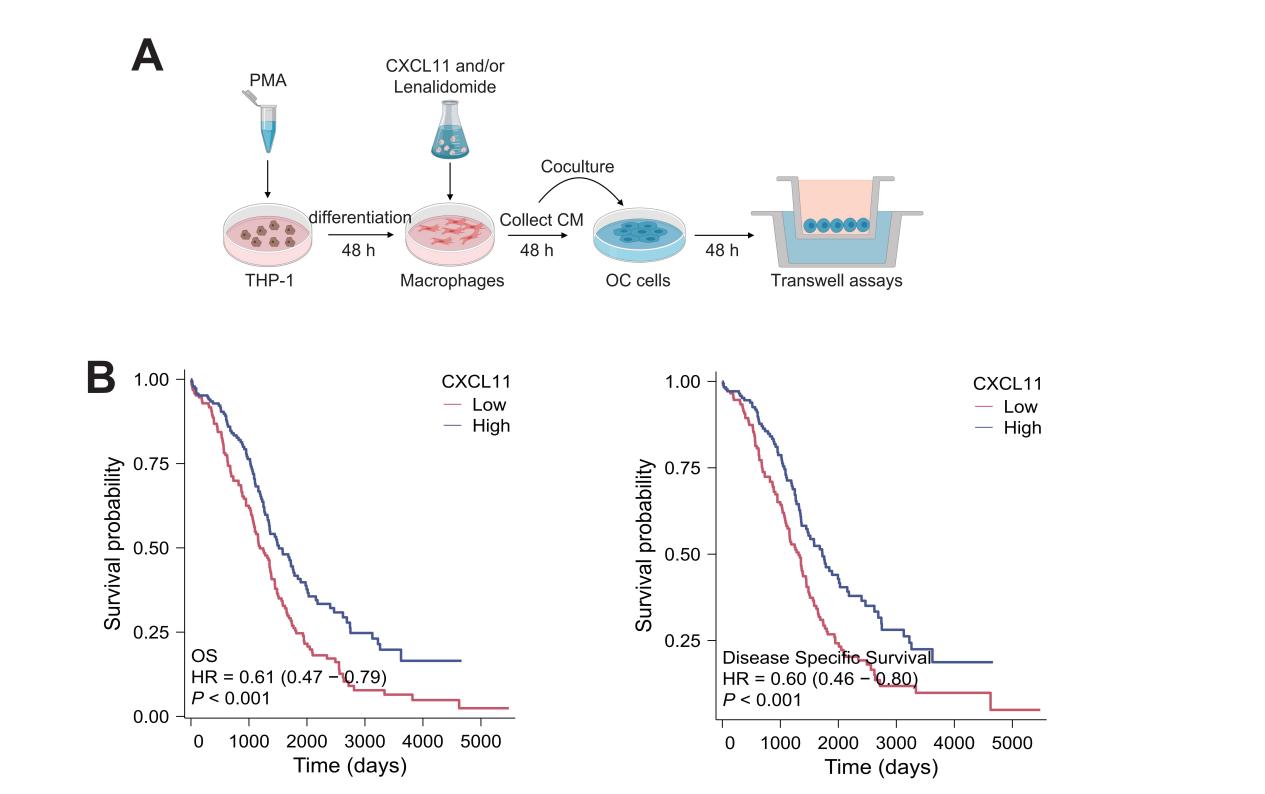


**Figure S5 CXCL11 is associated with favorable prognosis in EOC patients.**

(A) Mechanistic map of tumor cell migration and invasion under treatment with macrophage-CM. THP-1 cells were differentiated into macrophages by treatment with phorbol 12-myristate 13-acetate (PMA) for 48 h, followed by stimulation with recombinant CXCL11 protein and/or Lenalidomide for another 48 h. The CM were then collected and used to treat EOC cells for 48 h, after which the migratory and invasive abilities of the cells were evaluated using Transwell assays.

(B) The TCGA-OV datasets were analyzed to examine the effects of CXCL11 levels on OS and Disease Specific Survival of patients. Statistical analysis was performed using the log-rank test.
